# Choline supplementation for preterm infants: metabolism of four Deuterium-labeled choline compounds

**DOI:** 10.1007/s00394-022-03059-8

**Published:** 2022-12-03

**Authors:** Katrin A. Böckmann, Wolfgang Bernhard, Michaela Minarski, Anna Shunova, Cornelia Wiechers, Christian F. Poets, Axel R. Franz

**Affiliations:** 1 Department of Neonatology, Faculty of Medicine, Eberhard Karls University, Calwer Straße 7, 72076 Tuebingen, Germany; 2 Center for Pediatric Clinical Studies, Eberhard Karls University, Tübingen, Germany

**Keywords:** Deuterium, Stable isotope labeling, Choline, Supplementation, Preterm infant

## Abstract

**Background:**

Supply of choline is not guaranteed in current preterm infant nutrition. Choline serves in parenchyma formation by membrane phosphatidylcholine (PC), plasma transport of poly-unsaturated fatty acids (PUFA) via PC, and methylation processes via betaine. PUFA-PC concentrations are high in brain, liver and lung, and deficiency may result in developmental disorders. We compared different deuterated (D9-) choline components for kinetics of D9-choline, D9-betaine and D9-PC.

**Methods:**

Prospective study (1/2021–12/2021) in 32 enterally fed preterm infants (28 0/7–32 0/7 weeks gestation). Patients were randomized to receive enterally a single dose of 2.7 mg/kg D9-choline-equivalent as D9-choline chloride, D9-phosphoryl-choline, D9-glycerophosphorylcholine (D9-GPC) or D9-1-palmitoyl-2-oleoyl-PC(D9-POPC), followed by blood sampling at 1 + 24 h or 12 + 60 h after administration. Plasma concentrations were analyzed by tandem mass spectrometry. Results are expressed as median (25th/75th percentile).

**Results:**

At 1 h, plasma D9-choline was 1.8 (0.9/2.2) µmol/L, 1.3 (0.9/1.5) µmol/L and 1.2 (0.7/1.4) µmol/L for D9-choline chloride, D9-GPC and D9-phosphoryl-choline, respectively. D9-POPC did not result in plasma D9-choline. Plasma D9-betaine was maximal at 12 h, with lowest concentrations after D9-POPC. Maximum plasma D9-PC values at 12 h were the highest after D9-POPC (14.4 (9.1/18.9) µmol/L), compared to the other components (D9-choline chloride: 8.1 [5.6/9.9] µmol/L; D9-GPC: 8.4 (6.2/10.3) µmol/L; D9-phosphoryl-choline: 9.8 (8.6/14.5) µmol/L). Predominance of D9-PC comprising linoleic, rather than oleic acid, indicated fatty-acyl remodeling of administered D9-POPC prior to systemic delivery.

**Conclusion:**

D9-Choline chloride, D9-GPC and D9-phosphoryl-choline equally increased plasma D9-choline and D9-betaine. D9-POPC shifted metabolism from D9-betaine to D9-PC. Combined supplementation of GPC and (PO) PC may be best suited to optimize choline supply in preterm infants. Due to fatty acid remodeling of (PO) PC during its assimilation, PUFA co-supplementation with (PO) PC may increase PUFA-delivery to critical organs. This study was registered (22.01.2020) at the Deutsches Register Klinischer Studien (DRKS) (German Register for Clinical Studies), DRKS00020502.

**Study registration:**

This study was registered at the Deutsches Register Klinischer Studien (DRKS) (German Register for Clinical Studies), DRKS00020502.

**Supplementary Information:**

The online version contains supplementary material available at 10.1007/s00394-022-03059-8.

## Introduction

The supply of the essential nutrient choline is often insufficient, especially in preterm infants [1–3]. Choline is required as constituent of phosphatidylcholine (PC) and sphingomyelin (SPH) in all cell membranes, and in many secretions like bile and surfactant. In the form of PC, it is also required for the transport of the long-chain poly-unsaturated fatty acids (PUFA), namely docosahexaenoic acid (DHA) and arachidonic acid (ARA), via lipoproteins. Choline is required for neurodevelopment via synthesis of the neurotransmitter acetylcholine (ACh) [1, 4], and, via its oxidation product betaine, it is an important methyl group donor for the regeneration of methionine from homocysteine and essential methylation processes [5]. When choline is not completely absorbed in the small intestine, trimethylamine (TMA) is formed as a bacterial degradation product in the colon, depending on the intestinal microbiota. Following absorption, trimethylamine is oxidized by the liver to trimethylamine oxide (TMAO), which may be associated with cardiovascular events [3, 6–8]. In preterm infants, this seems to be less important, because enzymatic oxidation of trimethylamine is reduced due to immaturity [8].

Prenatally, choline is actively transported to the fetus via the placenta, resulting in high plasma-free choline concentrations of ⁓ 40 µmol/L [9]. We showed that plasma levels rapidly decrease by 50% or even more of fetal values after preterm birth [10]. This reduction in plasma concentrations is slower and occurs at a later developmental stage in term infants, characterized by a lower growth rate [11]. Due to preterm birth, the high level of continuous active trans-placental choline supply is interrupted in a phase of exponential general and brain growth and development (24–32 week postmenstrual age). Moreover, the fetus is exposed to and swallows ⁓ 400 mL/d-amniotic fluid comprising ⁓ 90 µM choline compounds, mainly PC, choline, SPH and *α*-glycerophosphorylcholine (GPC) ([25] and unpublished own analyses).

During the third trimester, volume of parenchyma, being rich in PC/SPH, increases. Particularly, brain gray matter becomes enriched in PUFA-PC [12, 13]. However, when choline levels in plasma and hepatic PC pools are low, choline is mobilized from other organs, particularly the lung [14]. In cystic fibrosis (CF) patients, low free choline levels were associated with impaired liver and lung function [3, 15]. Hence, due to this lung-to-liver drainage of choline in choline-deficient states, insufficient choline supply could also be a contributor to the multi-factorial pathogenesis of chronic lung disease of prematurity.

The US Food and Drug Administration defined choline as an essential nutrient in 1998 and adequate intakes (AI) were defined by the National Academy of Medicine of the USA (NAM) and the European Food Safety Authority (EFSA). The requirements are defined by age, sex, life conditions and genetics, with tightly defined AI and high upper tolerable limit (UL) values [16–18]. The European Society of the Paediatric Gastroenterology, Hepatology, and Nutrition Committee on Nutrition (ESPGHAN) recommended (2010) an enteral choline supply of 8–55 mg/kg/day for preterm infants, representing the AI and UL values of adults [19]. However, only the UL of this latter recommendation, i.e., enteral intakes of 50–60 mg/kg/day choline equivalent in postnatal preterm infants, resulted in plasma choline concentrations similar to those observed in umbilical cord plasma (which may represent fetal concentrations). [20].

Total choline intake through breastmilk and current formulas is lower than prenatal supply. Choline content of breast milk fortifiers varies greatly, resulting in infants’ plasma-free choline concentrations being only half those of the fetus, and even decreasing postnatally [21]. Estimated AI of choline is neither achieved by most formulas, frequently comprising free choline or PC, nor by breast milk predominantly containing phosphoryl-choline and GPC, together with PC and SPH [21].

In this study, we aimed to investigate potential differences in the plasma kinetics of choline, betaine and PC following supplementation of ‘free’ choline as a salt compared to its major physiologic water-soluble and lipophilic organic esters. We used their stable-isotope-labeled derivatives, namely D9-choline chloride, D9-phosphoryl-choline, D9-GPC and D9-POPC. This was to inform future enteral choline supplementation in preterm infants’ nutrition.

## Methods

This is a randomized parallel-group comparison in 32 preterm infants, carried out at Tübingen University Hospital, Germany. Patients were recruited from January 2021 to December 2021. The Institutional Review Board (project number 322/2019BO1) approved the protocol, and written informed consent was obtained prior to enrollment.

### Inclusion criteria

Gestational age at birth of 28 0/7–32 0/7 weeks and enteral intake ≥ 150 ml/kg/d.

### Exclusion criteria

Acute illness, diseases concerning the lipid metabolism especially of the gastrointestinal tract (enterostomy, pancreatic insufficiency, cholestasis), systemic therapy with corticosteroids, renal insufficiency (creatinine > 1.5 mg/dl), significant left or right ventricular failure with liver congestion, or missing consent.

### Supplementation

The Deuterium-labeled choline analogs D9-choline chloride ([*N*,*N*,*N*-trimethyl-d9] choline chloride), D9-phosphoryl-choline ([*N*,*N*,*N*-trimethyl-d9]phosphoryl-choline chloride, calcium salt), D9-GPC (L-alpha glycerylphosphoryl [*N*,*N*,*N*-trimethyl-d9]choline) and D9-POPC (rac-1-palmitoyl-2-oleoyl-SN-glycero-3-phosphoryl-choline-[*N*,*N*,*N*-trimethyl-d9]) were purchased as dry powder from EQ Laboratories GmbH (Augsburg, Germany). Doses were aliquoted, packaged in glass bottles and labeled by Rainfarn pharmacy (RAINFARN Gesundheit, Munich, Germany). For enteral application, the substances were reconstituted (i.e., dissolved or emulsified) in sterile water directly before administration.

### Study procedure

Using sealed opaque envelopes, patients were randomly allocated to the 4 different preparations:

- D9-choline chloride,
- D9-phosphoryl-choline chloride,
- D9-GPC or
- D9-POPC

Supplements were dissolved/emulsified in sterile water and 2 ml/kg (2.7 mg/kg or 24 µmol/kg D9 choline-equivalent) was given with a milk feed.

Blood samples (in total < 0.5 ml EDTA blood) were taken as randomly assigned at either 1 h and 24 h or at 12 h and 60 h after intake. Blood was immediately centrifuged at 1000x*g* at room temperature for 10 min. Plasma was separated and stored at − 80 °C until analysis.

### Chemical analysis

Blood plasma (50 µL) was extracted according to Bligh and Dyer [22]. Diarachidoyl-PC (PC20:0/20:0) and D_4_-choline chloride were used as internal standards. After centrifugation, the lower chloroform and the upper water:methanol phase were stored at -80 °C, and analyzed for (D9-)PC molecular species, and for (D9-)choline and its water-soluble metabolites, respectively, with LC-H-ESI-MS/MS as described before [13].

### Statistics

Differences in plasma kinetics or metabolic rates between these choline derivatives in preterm infants were unknown prior to the start of the study. Calculation of sample size was therefore based on plasma choline levels of preterm infants with and without choline supplementation. Plasma-free choline levels were 18.4 ± 4.8 µmol/L without and 37.7 ± 11.0 µmol/L with 30 mg/kg/d choline (choline chloride) supplementation for 10 days [20]. Assuming normal distribution of the data, a similar effect size and α ≤ 0.05, five individuals in each group (4 supplements) had to be analyzed to verify this clinically relevant impact on choline concentration with a power of 80%. Since these data were only indications but not a reliable basis for a sample size calculation/power analysis, 8 patients were allocated to each supplement (total of 32). All supplement groups were divided in 2 groups with 4 patients each. Blood samples were taken either at 1 and 24 h or at 12 and 60 h (also see graph 1). AUCs were calculated for each patient based on the trapezoidal rule (calculation of AUC is depicted in supplemental Figures s1 and s2). The primary outcome was defined as the area under the concentration curve (AUC) of D9-PC enrichment. Pre-defined secondary outcome variables were the maximum plasma concentration of D9-choline, D9-betaine and D9-PC and the distribution between the components over time. The term ‘total PC’, is referring to the sum of all measured molecular PC species.

Statistical and graphical analyses were done using JMP 14 (SAS Institute GmbH, Germany), and Excel 2010 (Microsoft Corporation, USA). Normal distribution was tested with Shapiro–Wilk test. Because several parameters were not normally distributed, results are shown as median with 25th and 75th percentile and non-parametric tests (Wilcoxon test) were used for between supplement comparisons.

A significance level of *p* = 0.05 was applied and we did not correct for multiple testing in this exploratory analysis. Standard Deviation Scores for weight (SDS) were calculated using LMSgrowth (version 2.14; http://www.healthforallchildren.com/?product=lmsgrowth) based on the British 1990 growth reference [23].

## Results

**Fig.1:**
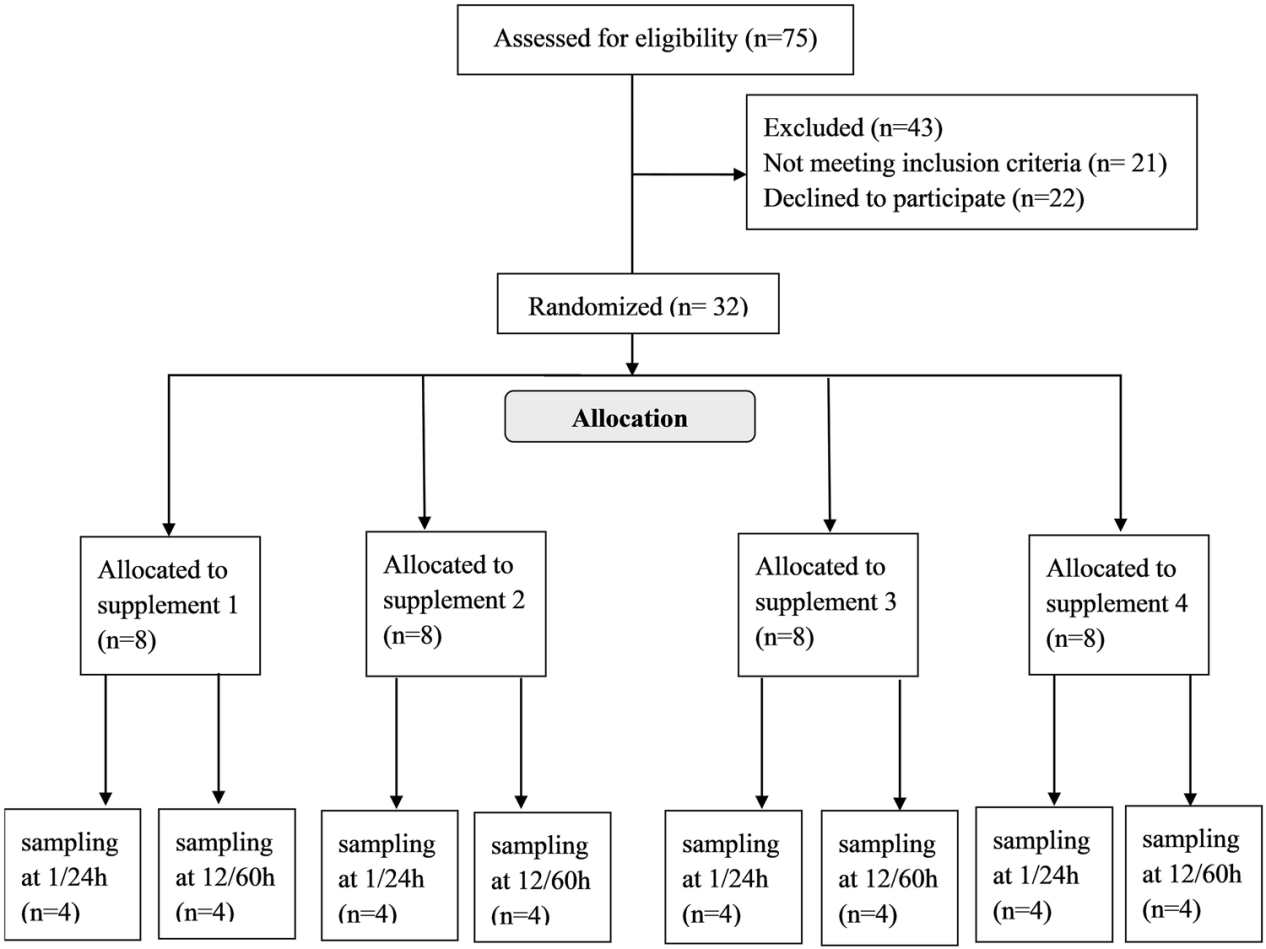
Participant Flow. *Supplement 1* D9-choline chloride, *supplement 2* D9-phosphoryl-choline, *supplement *3 D9-alpha-glycerophosphorylcholine, *supplement *4 D9-palmitoyl-oleoyl-Phosphatidylcholine, *h* hour, *n* number

Patient flow and demographics are shown in Fig. 1 and Table 1. There were no significant differences in weight (*p* = 0.65) or gestational age (*p* = 0.83) at birth between groups, and all patients completed the study.

**Table 1:** Demographic data

		Total	Choline chloride	Phosphoryl choline	GPC	POPC
Patients		32	8	8	8	8
Male/female		19/13	4/4	5/3	4/4	6/2
Gestational age at birth in weeks		30.2 (28.7/31.3)	30.0 (28.3/31.7)	29.9 (28.4/31.3)	30.7 (29.0/31.6)	29.9 (28.8/31.2)
Postnatal age at supplement (days)		14 (12/15)	13.5 (12/15)	14 (12.3/16.3)	14 (11.5/15.5)	13 (12.3/15)
Weight at birth (g)		1332 (1120/1605)	1450 (1265/1630)	1340 (1089/1783)	1267 (1071/1440)	1338 (1026/1508)
SDS birthweight		− 0.39 (− 1.33/0.37)	0.19 (− 0.39/1)	0 (− 1.24/0.67)	− 0.72 (− 1.53/− 0.47)	− 0.56 (− 1.57/0.37)
Weight at supplement (g)		1590 (1290/1760)	1640 (1310/1935)	1430 (1259/1727)	1460 (1335/1806)	1600 (1243/1670)
SDS weight at supplement		− 0.76 (− 1.4/− 0.15)	− 0.36 (− 0.77/0.32)	− 0.58 (− 1.12/− 0.29)	− 1.21 (− 1.68/− 0.90)	− 0.98 (− 1.83/-0.07)
Nutrition at supplement	Breast milk	19	5	6	4	4
	Breast milk + human donor milk	1	0	0	0	1
	Breast milk + formula	11	2	2	4	3
	Formula	1	1	0	0	0
CPAP at supplement		23	5	7	5	6
Infants with prenatal corticosteroids		29	7	7	7	8
Antibiotic treatment before/at supplement		13	4	2	4	3

Data are shown as median (Quartile 1/Quartile 3)

Every patient receiving breast milk or human donor milk, received FM85% (cow milk-based multicomponent human milk fortifier, Nestle, Frankfurt, Germany) with 4 g/100 ml in each meal

### D9-choline plasma concentrations

**Fig. 2:**
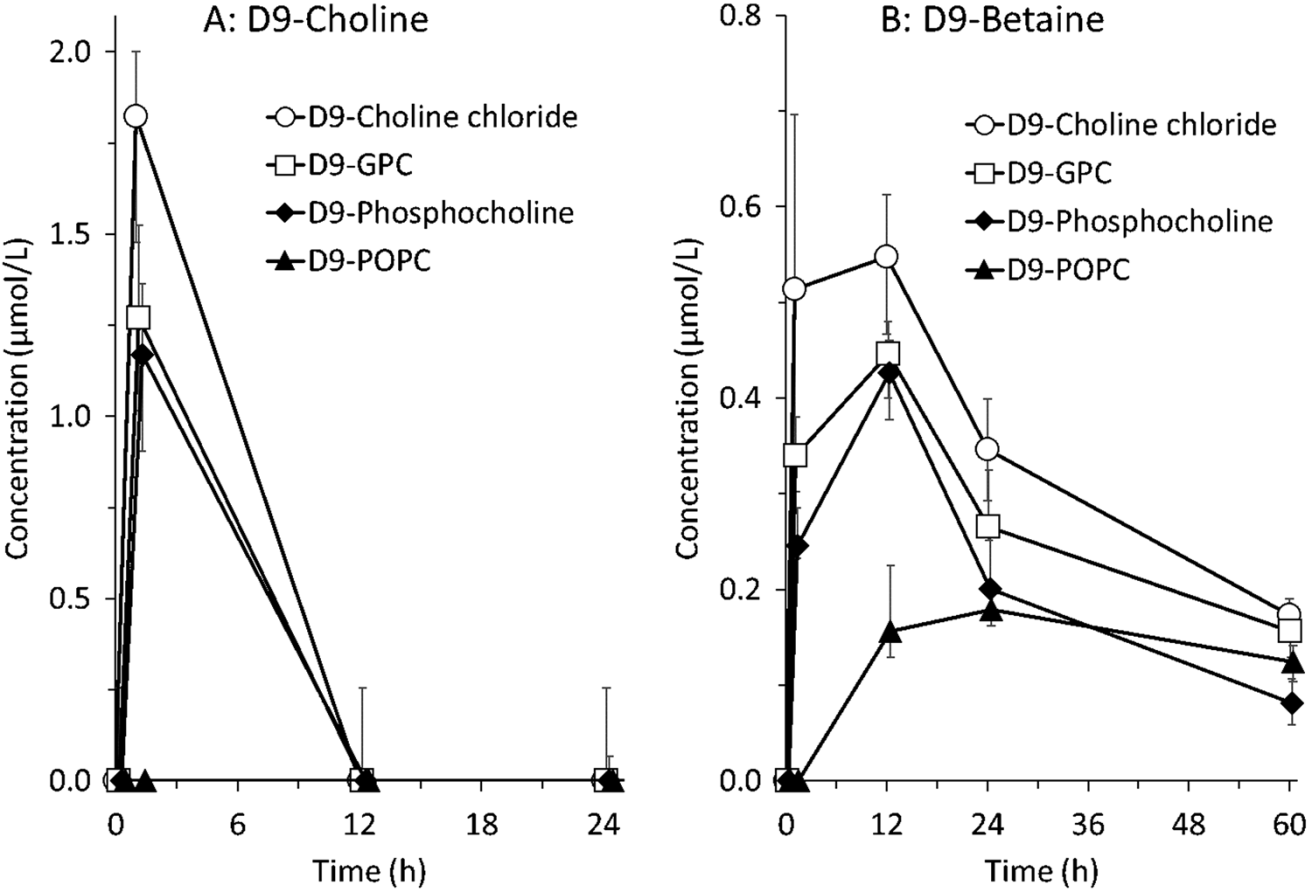
D9-Choline (**A**) and D9-betaine (**B**) concentrations in plasma following enteral administration of D9-labeled choline supplements (D9-choline chloride, D9-phosphoryl-choline, D9-alpha-glycerophosphorylcholine, D9-palmitoyl-oleoyl-Phosphatidylcholine) in 32 preterm infants. Symbols and bars indicate medians and interquartile ranges of 4 individual determinations. Abbreviations: *h* hours

Figure 2A shows D9-choline plasma concentrations at the measured time points in response to the intake of supplements. Concentrations rapidly increased after the intake of water-soluble compounds, with maximum levels measured at 1 h of 1.8 (0.9/2.2) µmol/L (D9-choline chloride), 1.3 (0.9/1.5) µmol/L (D9-GPC), and 1.2 (0.7/1.4) µmol/L (D9-phosphoryl-choline). After 12 h, D9-choline was no longer detectable in plasma. However, at the measured time points, there was no D9-choline detectable in plasma after intake of D9-POPC. There was a significant difference between the D9-choline plasma concentrations at 1 h after the intake between the 4 different supplements (*p* = 0.02). However, when comparing the plasma choline concentration at 1 h after intake of the three water-soluble (choline chloride, GPC and phosphoryl-choline) preparations, there was no significant difference (*p* = 0.3). Individual and mean plasma concentrations of native and D9-labeled choline and PC are provided in supplemental Table s1.

### D9-betaine plasma concentrations

D9-betaine plasma concentrations are shown in Fig. 2B. They showed a delayed kinetic compared to that of D9-choline, with rapid increase and maximal values at 12 h rather than 1 h. In contrast to D9-choline, D9-betaine was still present in plasma at 60 h. Notably, differences in D9-betaine plasma concentrations between water-soluble D9-choline components were present only at 1 h (*p* = 0.014), with highest values in response to D9-choline chloride, followed by D9-GPC and D9-posphorylcholine, but no significant difference at 12 h (*p* = 0.06). By contrast, after the administration of D9-POPC, D9-betaine was undetectable at 1 h. It exhibited the lowest and most delayed increase, with maximum values at 24 h, when D9-betaine concentrations following water-soluble components already had decreased.

### D9-TMAO plasma concentrations

Unlabeled TMAO was measured with low plasma concentrations in all groups (D9-choline chloride: 0.19 (0.12/0.38) µmol/l; D9-GPC: 0.11 (0.05/0.23) µmol/l, phosphoryl-choline: 0.09 (0/0.5) µmol/l; POPC 0.24 (0.03/0.35) µmol/l). However, D9-TMAO was not detected at any time point following all four D9-labeled choline compounds.

### D9-PC

**Fig.3:**
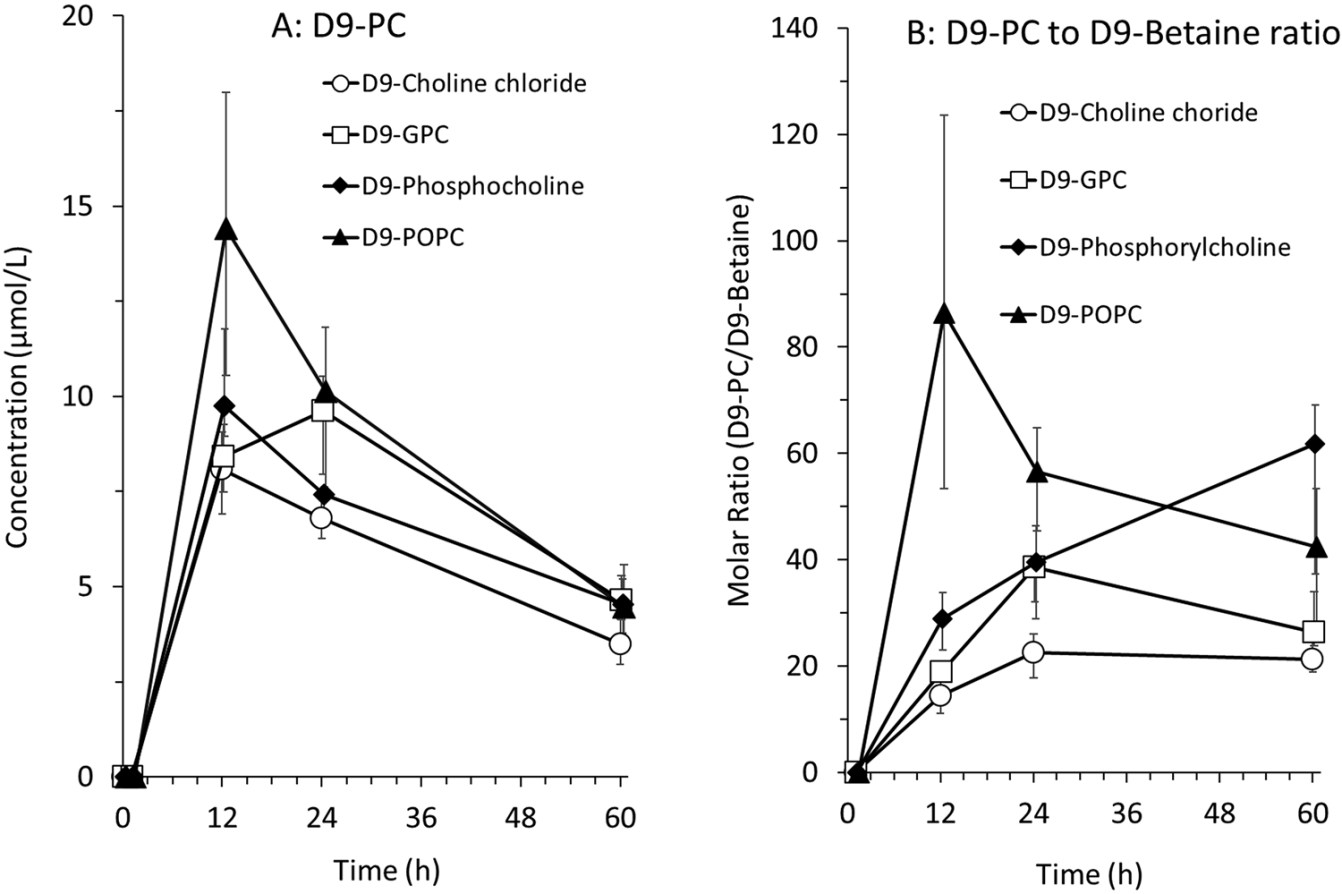
D9-phosphatidylcholine (PC) concentrations (**A**) and D9-PC to D9-betaine ratio (**B**) in plasma after the intake of D9-labeled choline supplements (D9-choline chloride, D9-phosphoryl-choline, D9-alpha-glycerophosphorylcholine, D9-palmitoyl-oleoyl-Phosphatidylcholine) in 32 preterm infants over time. Symbols and bars indicate medians and interquartile ranges of 4 individual determinations. Abbreviations: *h* hours

The time course of D9-PC (i.e., the sum of all measured molecular D9-PC species) concentrations in plasma is shown in Fig. 3A. D9-PC increased later than D9-choline, with maximum values measured at 12 h. Notably, following D9-POPC administration, the maximum concentration of D9-PC was the highest [14.4 (9.1/18.9) µmol/L], compared to 8.1 (5.6/9.9) µmol/L (D9-choline chloride), 8.4 (6.2/10.3) µmol/L (D9-GPC) and 9.8 (8.6/14.5) µmol/L (D9-phosphoryl-choline). These differences, however, did not reach significance (*p* = 0.14), nor did the differences in the AUC of D9-PC enrichment between the four supplements (D9-choline chloride: 15.5 (12.3/23.1) μmol/l × h, D9-GPC: 21.9 (19.6/25.8) µmol/l × h, D9-phosphoryl-choline: 17.5 (13.5/21.7) μmol/l × h; D9-POPC: 19.5 (11.7/30.5) μmol/l × h; *p* = 0.38). When comparing each group separately, there were also no significant differences.

By contrast, the D9-PC to D9-betaine ratio was higher for D9-POPC compared to the water-soluble D9-choline compounds at 12 h, indicating preferential D9-PC formation at the expense of D9-betaine formation following D9-POPC (Fig. 3B). Irrespective of the D9-tracer used, beyond 12 h plasma D9-PC showed a similarly delayed decrease as D9-betaine.

### Effects of D9-choline supplements on fatty acid metabolism of D9 PC

**Fig.4:**
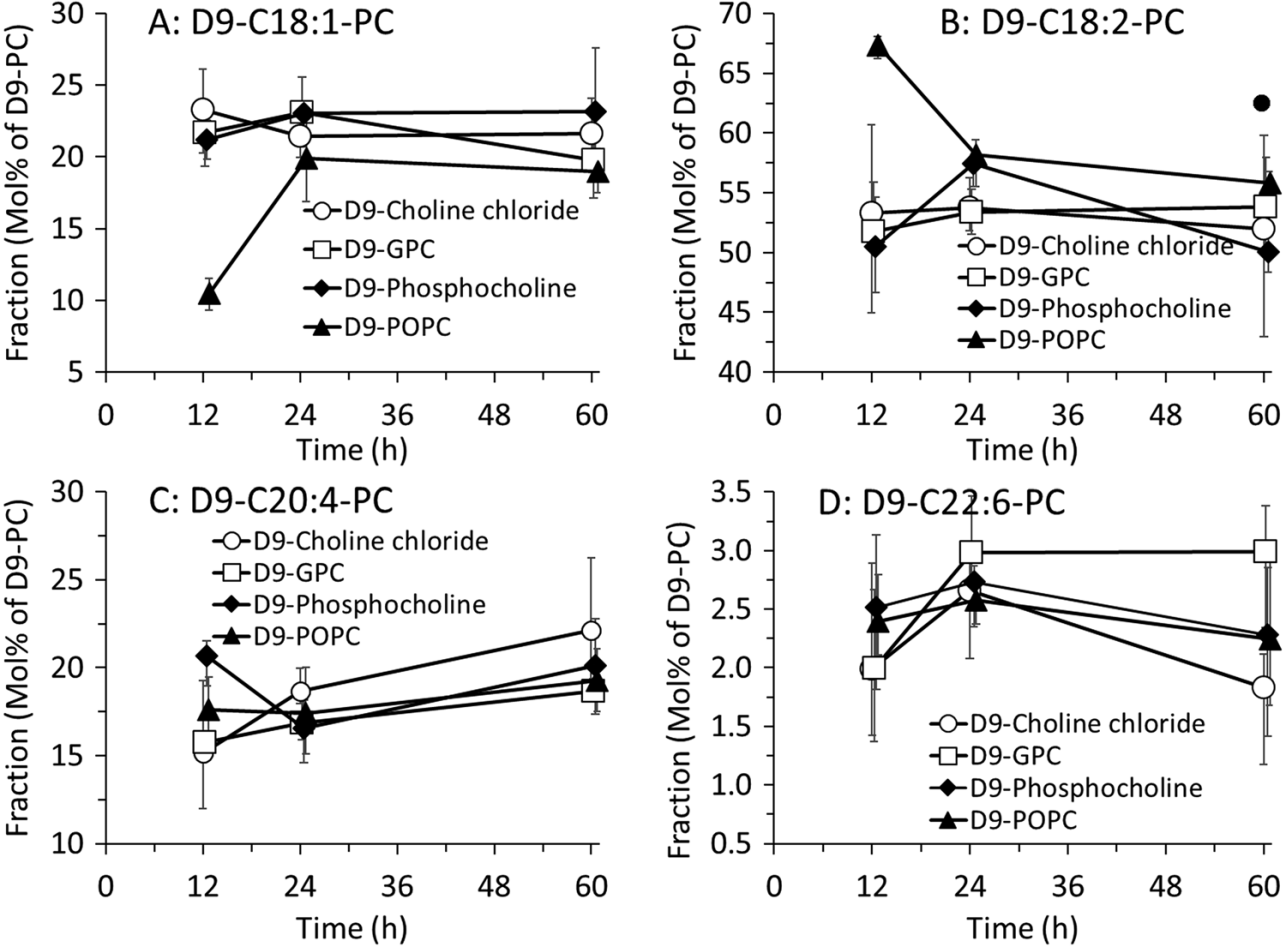
Molar fraction kinetics of D9- phosphatidylcholine (PC) in response to different D9-choline supplements. D9-PC molecular species were grouped according to their content in an oleic acid (D9-C18:1-PC) (**A**), linoleic (D9-C18:2-PC) (**B**), arachidonic acid (D9-C20:4-PC) (**C**) or docosahexaenoic acid residue (D9-C22:6-PC) (**D**), and expressed as fractions of total D9-PC (total D9-PC = 100%). Symbols represent medians and bars interquartile ranges

As PC metabolism and its fatty acid content are organotypic and show characteristic kinetics, we differentiated D9-PC into the major PC sub-groups of plasma as previously demonstrated [24] (Fig. 4). In brief, such differentiation is due to the fact that the fatty acid residue in position 1 of the glycerol backbone of PC is mostly saturated (palmitic or stearic acid), whereas the other one is unsaturated. Hence, we differentiated D9-PC based on the unsaturated fatty acid in position 2 into those comprising predominantly an oleic acid (OA) or other less common fatty acids containing also 18 carbon units and 1 double bond (i.e., C18:1) (D9-C18:1-PC) (Fig. 4A), linoleic acid (= C18:2, LA) (D9-C18:2-PC) (Fig. 4B), arachidonic acid (= C20:4, ARA) (D9-C20:4-PC) (Fig. 4C) or docosahexaenoic acid (C22:6, DHA) (D9-C22:6-PC) (Fig. 4D). Our data show the general predominance of D9-PC containing LA (C18:2), followed by compounds comprising OA (C18:1) or ARA (C20:4) (Fig. 4), whereas DHA-containing D9-PC was a minor component in these patients.

Notably, D9-PC containing oleic acid (D9-C18:1-PC, Fig. 4A) was lowest after D9-POPC, although this supplement belongs to the D9-C18:1-PC subgroup. By contrast, in response to D9-POPC, components containing linoleic acid (D9-C18:2-PC, Fig. 4B) or arachidonic acid (D9-C20:4-PC, Fig. 4C) were initially higher in response to D9-POPC. Beyond 12 h after D9-choline administration, molar fractions of D9-PC sub-groups approached similar values for all D9-choline supplements (Fig. 4A–D). D9-lyso-PC reached maximal concentration after 12 h after the administration of D9-choline chloride, D9-phosphoryl-choline and highest after D9-POPC, whereas after D9-GPC maximum values were reached after 24 h. After 60 h, D9-lyso-PC was still detected in response to D9-choline chloride, D9-phosphorylchloride and D9-GPC (Fig. s3).

### Correlation labeled and unlabeled metabolites

There were weak correlations between D9-choline plasma concentration at 1 h and native choline concentration for the water-soluble supplements (Supplemental Figure s4 A) and D9-PC and native PC concentrations in plasma (Supplemental Figure s4 B).

## Discussion

In this study, we investigated the metabolism of four different choline compounds, which all might be suitable to optimize choline supplementation of very preterm infants. To better follow the differences in the kinetics of individual compounds, we applied stable isotope labeling of the tri-methyl-ammonium group of the choline unit with Deuterium (D9-choline) of supplements, as previously used for choline chloride only [3, 20]. This study was limited by ethical restrictions in infants, namely with respect to the number of venous punctures in this highly vulnerable population. Very sparse blood sampling was carried out (only 2 samples per patient), to avoid venipunctures in addition to those being clinically indicated. Consequently, exact kinetics and time points of maximum concentrations could not be determined. Hence, results need to be considered in the context of previous and future reports on (D9-labeled) choline compounds enterally administered to adults, where more frequent blood sampling is possible [3, 15, 25]. In this study on very preterm infants, enteral administration of any water-soluble D9-labeled choline compound used resulted in the rapid appearance of D9-choline and D9-betaine in plasma, suggesting that they all are rapidly absorbed, and that a significant portion is metabolized to D9-betaine. D9-choline was absent from plasma at 12 h and beyond, suggesting complete cellular uptake and metabolism. However, the maximum time point and half-life (t1/2) of free plasma D9-choline, that is 1–2 h in adults [3, 15], could not be assessed in these preterm because the number of blood samples was limited to two for ethical considerations. Although choline is released into plasma from degraded PC in neonatal rats, this was not measurable here, likely due to the lower D9-choline label in human studies compared to animal experiments (2.7 vs. 37.5 mg/kg) [2]. Rapid increase of D9-betaine at 1 h further accentuates the rapid assimilation and metabolism of water-soluble D9-choline compounds. It is important to note, however, that D9-betaine maximum values were at 12 h rather than 1 h as determined for D9-choline, and that values had not returned to zero at 60 h. This indicates that a single choline administration, although not having a sustainable effect on plasma choline, increases the pool of betaine as a methyl donor for a longer period.

Following D9-POPC administration, however, D9-choline was not detected at any time point. D9-betaine was detected only at 12–60 h, but not at 1 h, and D9-betaine maximum plasma concentrations were lower compared to water-soluble compounds. This is important for three reasons: first, betaine is synthesized from free choline by choline dehydrogenase and betaine-aldehyde dehydrogenase [26]. Hence, part of D9-POPC must have been hydrolyzed to free D9-choline, but did not enter the circulation at all, or did so between 1 and 12 h following D9-POPC administration. Second, as D9-betaine increased later, with zero values at 1 h, D9-choline release from D9-POPC was delayed. This is consistent with previous data in adults, where water-soluble compounds resulted in choline maxima at 1–2 h, while choline concentrations following egg-PC levels peaked at 3 h [25]. Third, these data support that intestinal choline metabolism is different for PC compared to its water-soluble counterparts in preterm infants as previously described in adults: free choline from choline chloride is absorbed and released to the portal vein, whereas the precise mechanisms of phosphoryl-choline and GPC absorption and enterocytic metabolism are still unclear, but its choline moiety apparently enters the portal vein as well. (PO)PC, however, is initially hydrolyzed by secretory pancreatic phospholipase A2IB (pPLaseA2IB), followed by the absorption of lyso-PC and fatty acid. About 50% of lyso-PC is then resynthesized to PC for chylomicron assembly and lymphatic transport bypassing the liver, using a fatty acid (acyl-coenzyme A) within the enterocyte, whereas only the remaining 50% are transported to the liver via the portal vein [26, 27]. This re-acylation occurs by enterocytic lyso-PC acyl transferase 3 (LPCAT3), being important for plasma lipid transport and favoring incorporation of poly-unsaturated fatty acids [28]. In line with these mechanisms of choline assimilation from PC, and the involvement of pPLaseA2IB and enterocytic LPCAT3, preserving ingested D9-POPC from complete degradation and D9-choline release, D9-POPC resulted in the highest D9-PC/D9-betaine ratio in these preterm infants. Consequently, (PO)PC administration favors PC formation at the expense of choline oxidation to betaine. As betaine increased to values above fetal and neonatal levels upon choline supplementation in preterm infants [20], it may be adequate to use PC as a choline supplement in combination with water-soluble compounds. However, it must be considered that some preterm infants appear to have exocrine pancreatic insufficiency [29, 30], which may impair cleavage of PC and reduce its uptake as lyso-PC; hence, the effectiveness of choline supplementation using PC. This could be especially important in very preterm infants, which would be the population with the greatest need for choline supplementation.

The concept of PC supplementation in preterm infants may additionally be useful with respect to the de-acylation/re-acylation process that PC undergoes during assimilation. We have previously shown that following preterm birth, linoleic acid-containing PC (C18:2-PC) increases at the expense of compounds comprising arachidonic (C20:4-PC) or docosahexaenoic acid (C22:6-PC). This is due to the low supply with these fatty acids and high supply with linoleic acid from breast milk and formula, which is in contrast to placental retention of linoleic acid and preferential arachidonic and docosahexaenoic acid delivery to the fetus [31]. In line with this, D9-POPC was rapidly converted to linoleic acid-containing D9-C18:2-PC, with low yields of D9-C20:4-PC and D9-C22:6-PC. It is reasonable to expect, but must be tested in future experiments, that higher dietary administration of arachidonic and docosahexaenoic acid in combination with PC as a choline supplement, at the expense of linoleic acid supply, will result in PUFA-PC composition that resembles fetal plasma PC profile [32].

Finally, experiments in adults have shown that TMAO is formed from water-soluble choline supplements but not from PC [33]. In these preterm infants, D9-TMAO was not detected in plasma in response to any (D9-) choline compounds, which confirms previous data [20]. Unlabeled TMAO was present in plasma of all infants, but concentrations were about 1/10 of those observed in fasting plasma samples of healthy adults [25]. This may be due to the intestinal microbiome of predominantly breast milk-fed infants or due to the immaturity of their liver, being not yet able to oxidize trimethylamine to TMAO [8]. Nevertheless, administered free choline may undergo intestinal bacterial degradation without being represented by increased TMAO, as a result of small intestinal bacterial overgrowth (SIBO) in intestinally compromised patients [27].

Our findings are important to optimize future preterm infant nutrition. Assuming that the prenatal choline plasma concentrations are optimal for rapid fetal growth and development, we previously showed that an enteral supply of 50–60 mg/kg/d choline is needed to achieve these prenatal choline plasma concentrations in enterally fed preterm infants [20]. Breast milk or many preterm infant formulae do not meet these requirements, resulting in the pertinence of low plasma choline [1, 10, 21]. Since 2010, the ESPGHAN recommends an enteral choline supply of 8–55 mg/kg/day to very preterm infants [19]. Our data have shown that the upper limit of this broad recommendation should be aimed for [20].

We detected similarly high D9-choline concentrations in plasma after supplementation with D9-choline chloride, D9-phosphoryl-choline and D9-GPC. Due to sparse sampling, it remains unclear whether the minor differences in maximal plasma concentrations at 1 h are clinically relevant and due to substance-specific differences in the absorption kinetics. Previous data from adults may point in this direction [25]: phosphoryl-choline and GPC, which are the major natural choline components in breast milk [21, 34, 35], showed a lower formation of TMAO in adults [25] and tended to result in a lower increase in plasma D9-betaine compared to choline chloride (see Fig. 2B). Since phosphoryl-choline is currently not commercially available as a supplement, we consider GPC the most suitable water-soluble organic choline ester to increase choline plasma concentrations in preterm infants. Although GPC has been used in infant formulas, without apparent adverse effects, its use is still an exception [21].

The observation that native choline concentrations weakly correlated with D9 choline plasma concentrations may indicate that there may be inter-individual differences in absorption and availability of enterally administered choline, which must be considered in future studies. The observation that the GPC group had lower native choline concentrations may indicate that we underestimate the effect of supplementation with GPC on choline plasma concentrations. It may have been of additional interest to determine exact daily baseline choline intake in the study subjects, to see whether native choline concentrations correlate with this intake.

Optimizing fatty acid composition of plasma PC is key to plasma transport and organ delivery of PUFA, such as arachidonic and docosahexaenoic acid [32]. This may be of particular importance for the developing brain and eye, where combined arachidonic and docosahexaenoic acid supplementation improved retinopathy outcome [36]. Docosahexaenoic acid is needed for synaptogenesis, membrane fluidity and retinal receptor activity [37], but arachidonic acid and its elongation product adrenic acid surmount docosahexaenoic acid in brain lipids [12]. Arachidonic acid is a precursor of eicosanoids and also needed for growth and cell signal systems [38–40]. Decreased levels are associated with neonatal morbidity [41]. It is of special interest that, while D9-POPC (18:1) was supplemented, predominantly D9-PC with linoleic acid (18:2) was measured in plasma, presumably because linoleic acid is the predominant poly-unsaturated fatty acid in western breast milk and infant formulas [31, 42]. In a previous study, we showed that in adipose tissue of term infants, studied immediately after birth, the proportion of arachidonic and docosahexaenoic acid was high in triglycerides and other lipids [43], because these are actively enriched in the fetus prenatally, whereas linoleic acid is retained in the mother [44]. However, in preterm infants at term (after about 10 weeks of feeding in the NICU), the adipose tissue content of arachidonic and docosahexaenoic acid was significantly lower while linoleic acid was greatly increased [43]. According to the processing of D9-POPC during its assimilation described here, co-supplementation of docosahexaenoic and arachidonic acid together with POPC in preterm infants could improve the non-physiologic molecular composition of plasma PC, and potentially help to improve long-term outcome in preterm infants by improving PUFA supply to the brain and other organs. It could also be an option to supply purified DHA-PC and ARA-PC, which can be found in milk fat globular membranes but, at present, these complex mixtures, with many additional compounds and variable content by batch, require further investigation.

## Conclusion

All water-soluble D9-choline supplements increased plasma levels of D9-choline and D9-betaine. Highest levels were detected after the intake of D9-choline chloride and D9-GPC, with D9-GPC showing lower D9-betaine formation. Since GPC is a natural component of breast milk, we suggest this as the most suitable water-soluble choline supplement for preterm infants. Absence of plasma D9-choline after D9-POPC may be due to delayed absorption and different metabolism, as it increased plasma D9-PC at the expense of D9-betaine compared to water-soluble components. De- and re-acylation of administered PC as a choline source has the potential to shuttle arachidonic and docosahexaenoic acid into the PC-fraction of chylomicrons, with PC as their major transport form in plasma. (PO)PC also leads to lowest levels of betaine, which is strongly increased in response to water-soluble choline supplements. We therefore conclude that a combination of GPC and PC may be the most suitable choline supplement for preterm infants.

## Supplementary Information

Below is the link to the electronic supplementary material.

Supplementary file1 (DOCX 152 KB)

## Data Availability

Data supporting the findings of this study are available within the article and the online supplement.

## Abbreviations

ACh: Acetylcholine
AI: Adequate intake
ARA: Arachidonic acid
DHA: Docosahexaenoic acid
D9-: Molecules labeled with 9 Deuterium atoms at the trimethylammonium group
EDTA: Ethylene diaminetetra acetate
EFSA: European Food Safety Authority
ESPGHAN: European Society of the Paediatric Gastroenterology, Hepatology, and Nutrition Committee on Nutrition
GPC: Alpha-glycerophosphorylcholine
NAM: National Academy of Medicine of the USA
NICU: Neonatal intensive care unit
PC: Phosphatidylcholine
POPC: 1-Palmitoyl-2-oleoyl-PC (1-palmitoyl-2-oleoyl-glycero-3-phosphoryl-choline)
PUFA: Poly-unsaturated fatty acid
SPH: Sphingomyelin
TMAO: Trimethylamine oxide
