# Supplementary material for: Choline supplementation for preterm infants: metabolism of four Deuterium-labeled choline compounds
Source: Eur J Nutr. 2022 Dec 3;62(3):1195–205. doi: 10.1007/s00394-022-03059-8 (PMC10030424; doi:10.1007/s00394-022-03059-8)
Supplement: Supplementary file 1 — Supplementary file1 (DOCX 152 KB) [file 394_2022_3059_MOESM1_ESM.docx]

**Supplementary Information – European Journal of Nutrition**

**Choline supplementation for preterm infants - metabolism of four Deuterium-labeled choline compounds**

Katrin A. Böckmann^1^, Wolfgang Bernhard^1^, Michaela Minarski^1^, Anna Shunova^1^, Cornelia Wiechers^1^, Christian F. Poets^1^, Axel R. Franz^1, 2^

**Affiliations:** ^1^Department of Neonatology, Eberhard Karls University, Tübingen, Germany ^2^Center for Pediatric Clinical Studies, Eberhard Karls University, Tübingen, Germany

**Address of correspondence**: Katrin Alexandra Böckmann, MD, Department of Neonatology, Faculty of Medicine, Eberhard Karls University, Calwer Straße 7, D-72076 Tuebingen, Germany; katrin.boeckmann@med.uni-tuebingen.de; Phone: +49 7071 29 84742

| Patient ID | Supple-ment | Hours after Application | Native Choline | D9-Choline | Native PC | D9-PC |
| --- | --- | --- | --- | --- | --- | --- |
| 01 | **D9-Choline chloride** | 1 | 31.3 | 1.88 | 1313 | 0.00 |
| 10 |  | 1 | 19.3 | 0.60 | 1366 | 0.00 |
| 22 |  | 1 | 32.4 | 1.77 | 1548 | 0.00 |
| 25 |  | 1 | 30.5 | 2.37 | 1854 | 0.01 |
| 06 |  | 12 | 23.9 | 0.00 | 1643 | 10.35 |
| 09 |  | 12 | 21.3 | 0.00 | 1471 | 7.55 |
| 17 |  | 12 | 17.3 | 0.00 | 1985 | 8.62 |
| 26 |  | 12 | 27.8 | 0.00 | 1611 | 4.96 |
| 01 |  | 24 | 25.3 | 0.00 | 1404 | 6.38 |
| 10 |  | 24 | 22.2 | 0.00 | 1292 | 7.22 |
| 22 |  | 24 | 31.3 | 0.00 | 1654 | 8.40 |
| 25 |  | 24 | 29.9 | 0.00 | 1769 | 5.88 |
| 06 |  | 60 | 21.3 | 0.00 | 1400 | 4.67 |
| 09 |  | 60 | 18.7 | 0.00 | 1386 | 3.99 |
| 17 |  | 60 | 18.0 | 0.00 | 1929 | 2.81 |
| 26 |  | 60 | 23.9 | 0.00 | 1653 | 3.00 |
|  |  | Median (P25/P75) | **23.9 (20.8/30.1)** |  | **1579 (1396/1683)** |  |
| 04 | **D9-GPC** | 1 | 13.0 | 0.92 | 1272 | 0.00 |
| 16 |  | 1 | 15.9 | 1.03 | 1372 | 0.01 |
| 24 |  | 1 | 16.2 | 1.53 | 1421 | 0.01 |
| 31 |  | 1 | 21.1 | 1.51 | 1606 | 0.00 |
| 02 |  | 12 | 32.6 | 0.00 | 1683 | 10.82 |
| 14 |  | 12 | 15.5 | 0.00 | 1289 | 8.75 |
| 20 |  | 12 | 24.1 | 0.00 | 1879 | 8.11 |
| 27 |  | 12 | 19.8 | 0.00 | 1803 | 5.60 |
| 04 |  | 24 | 18.2 | 0.00 | 1303 | 10.28 |
| 16 |  | 24 | 9.0 | 0.00 | 1248 | 4.91 |
| 24 |  | 24 | 16.2 | 0.00 | 1403 | 8.96 |
| 31 |  | 24 | 19.4 | 0.00 | 1571 | 11.25 |
| 02 |  | 60 | 26.6 | 0.00 | 1722 | 5.94 |
| 14 |  | 60 | 13.9 | 0.00 | 1308 | 3.97 |
| 20 |  | 60 | 15.7 | 0.00 | 1901 | 4.19 |
| 27 |  | 60 | 29.6 | 0.00 | 1715 | 5.08 |
|  |  |  | **17.2 (15.6/21.8)** |  | **1496 (1307/1717)** |  |
| 05 | **D9-Phosphorylcholine** | 1 | 29.3 | 0.67 | 1460 | 0.00 |
| 15 |  | 1 | 25.0 | 0.98 | 1404 | 0.00 |
| 18 |  | 1 | 15.7 | 1.35 | 1357 | 0.00 |
| 28 |  | 1 | 21.7 | 1.39 | 1622 | 0.03 |
| 03 |  | 12 | 17.4 | 0.00 | 1893 | 15.89 |
| 11 |  | 12 | 37.1 | 0.13 | 1974 | 10.40 |
| 23 |  | 12 | 23.8 | 0.00 | 1635 | 9.11 |
| 32 |  | 12 | 18.7 | 0.00 | 1595 | 8.49 |
| 05 |  | 24 | 41.8 | 0.00 | 1651 | 7.43 |
| 15 |  | 24 | 23.7 | 0.00 | 1434 | 5.76 |
| 18 |  | 24 | 16.9 | 0.27 | 1619 | 7.41 |
| 28 |  | 24 | 16.6 | 0.00 | 1573 | 8.20 |
| 03 |  | 60 | 19.9 | 0.00 | 1767 | 6.45 |
| 11 |  | 60 | 28.3 | 0.00 | 1884 | 4.29 |
| 23 |  | 60 | 29.2 | 0.00 | 1464 | 2.21 |
| 32 |  | 60 | 15.4 | 0.00 | 1778 | 4.79 |
|  |  |  | **22.7 (17.3/28.5)** |  | **1621 (1463/1770)** |  |
| 08 | **D9-POPC** | 1 | 23.4 | 0.00 | 1467 | 0.00 |
| 13 |  | 1 | 15.5 | 0.00 | 1591 | 0.00 |
| 21 |  | 1 | 30.4 | 0.00 | 1620 | 0.00 |
| 29 |  | 1 | 21.0 | 0.00 | 1728 | 0.00 |
| 07 |  | 12 | 16.3 | 0.00 | 2066 | 11.30 |
| 12 |  | 12 | 22.9 | 0.00 | 1842 | 17.54 |
| 19 |  | 12 | 21.9 | 0.35 | 1707 | 8.30 |
| 30 |  | 12 | 28.6 | 0.00 | 1681 | 19.32 |
| 08 |  | 24 | 21.7 | 0.00 | 1468 | 3.77 |
| 13 |  | 24 | 19.1 | 0.00 | 1618 | 11.45 |
| 21 |  | 24 | 30.5 | 0.00 | 1660 | 8.81 |
| 29 |  | 24 | 23.1 | 0.00 | 1580 | 12.92 |
| 07 |  | 60 | 22.3 | 0.00 | 2042 | 3.94 |
| 12 |  | 60 | 22.5 | 0.00 | 1650 | 5.00 |
| 19 |  | 60 | 18.6 | 0.00 | 1449 | 1.49 |
| 30 |  | 60 | 17.0 | 0.00 | 1731 | 7.27 |
|  |  |  | **22.1 (18.9/23.2)** |  | **1655 (1589/1729)** |  |

Supplemental Table 1: Concentrations of native choline and phosphatidylcholine and their D9-labeled analogues of 64 measurements in 32 preterm infants. Samples were taken at 1h+24h or 12h+60h after administration of the D9-labeled supplement. Groups showed similar native choline concentrations, Wilcoxon for all groups p= 0.2, but significant difference between choline chloride and GPC group, p= 0.05. Native PC concentrations were also similar, Wilcoxon for all groups p= 0.4, also no significant difference when comparing each group. Abbreviation: D9: deuterium labeled, PC= phosphatidylcholine, D9-POPC= D9-palmitoyl-oleoyl-phosphatidylcholine,

Supplemental Figure s1; Method of AUC calculation.

AUC was calculated for each patient. When blood was taken at 1 and 24h an area under curve for 0, 1, 24 and 60h was calculated, assuming a value of 0 for the timepoint 0 and 60h.

Supplemental Figure s2; Method of AUC calculation

AUC was calculated for each patient. When blood was taken at 12 and 60h an area under curve for 0, 12 and 60h was calculated, assuming a value of 0 for the timepoint 0h.


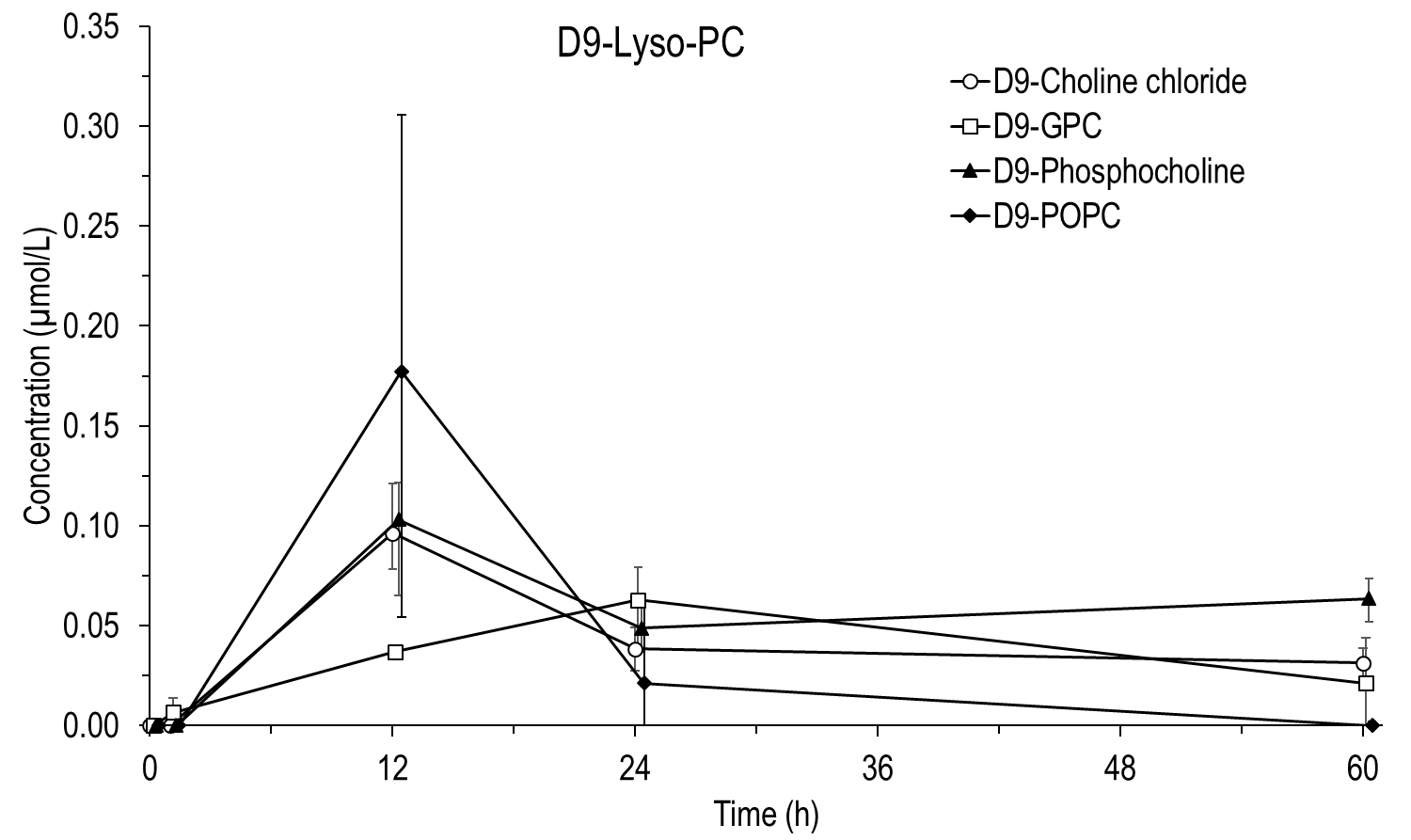


Supplemental Figure s3: D9-Lyso-PC concentrations in plasma after the administration of four deuterium labeled supplements**.**

Abbreviations: D9= deuterium labeled, D9-GPC= D9-alpha-glycerophosphorylcholine, PC= phosphatidylcholine, D9-POPC= D9-palmitoyl-oleoyl-phosphatidylcholine, h=hour.


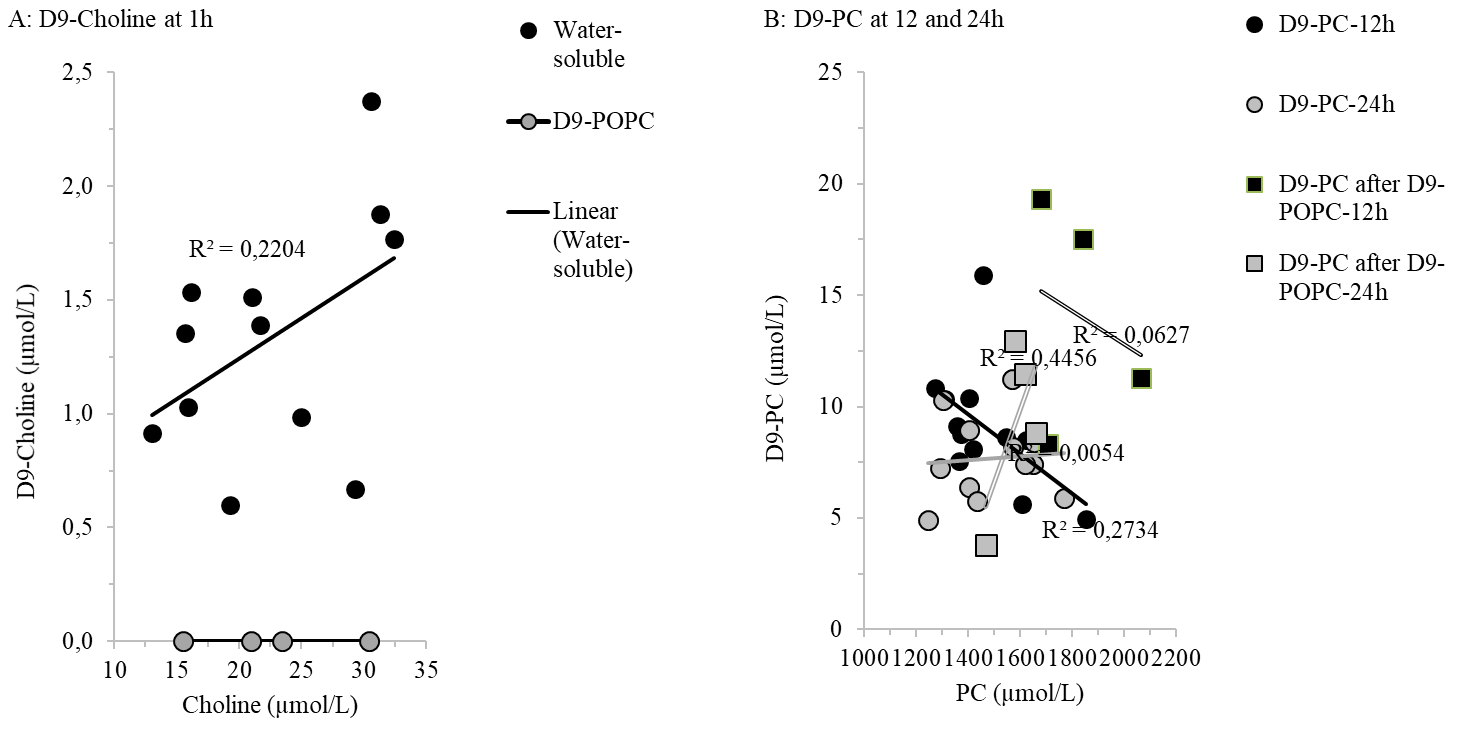


Supplementary Figure s4: Correlations of plasma concentrations of D9-choline relative to native choline at 1h (A) and of D9-PC relative to native PC at 12 and 24h after D9-choline supplementation (B) of 32 preterm study infants Abbreviations: h= hour, D9: deuterium labeled, PC= phosphatidylcholine, D9-POPC= D9-palmitoyl-oleoyl-phosphatidylcholine.
